# Licochalcone A Prevents the Loss of Dopaminergic Neurons by Inhibiting Microglial Activation in Lipopolysaccharide (LPS)-Induced Parkinson’s Disease Models

**DOI:** 10.3390/ijms18102043

**Published:** 2017-09-22

**Authors:** Bingxu Huang, Juxiong Liu, Chen Ju, Dongxue Yang, Guangxin Chen, Shiyao Xu, Yalong Zeng, Xuan Yan, Wei Wang, Dianfeng Liu, Shoupeng Fu

**Affiliations:** College of Animal Science and Veterinary Medicine, Jilin University, Changchun 130062, China; huangbingxu123@163.com (B.H.); juxiong@jlu.edu.cn (J.L.); 15754305616@163.com (C.J.); yangdxaig@163.com (D.Y.); gxchen51143@163.com (G.C.); xushiyao1989@163.com (S.X.); zengyalong@foxmail.com (Y.Z.); yanxuan1992@outlook.com (X.Y.); wangwei@jlu.edu.cn (W.W.); ccldf@163.com (D.L.)

**Keywords:** Licochalcone A, microglia, Parkinson’s disease, MAPK, NF-κB

## Abstract

The neuroprotective effects of Licochalcone A (Lico.A), a flavonoid isolated from the herb licorice, in Parkinson’s disease (PD) have not been elucidated. The prominent pathological feature of PD is the loss of dopaminergic neurons. The crucial role of neuroinflammation induced by activated microglia in dopaminergic neurodegeneration has been validated. In this study, we explore the therapeutic effects of Lico.A in lipopolysaccharide (LPS)-induced PD models in vivo and in vitro. We find that Lico.A significantly inhibits LPS-stimulated production of pro-inflammatory mediators and microglial activation by blocking the phosphorylation of extracellular signal-regulated kinase (ERK1/2) and nuclear factor κB (NF-κB) p65 in BV-2 cells. In addition, through cultured primary mesencephalic neuron-glia cell experiments, we illustrate that Lico.A attenuates the decrease in [^3^H] dopamine (DA) uptake and the loss of tyrosine hydroxylase-immunoreactive (TH-ir) neurons in LPS-induced PD models in vitro. Furthermore, LPS intoxication in rats results in microglial activation, dopaminergic neurodegeneration and significant behavioral deficits in vivo. Lico.A treatment prevents microglial activation and reduction of dopaminergic neuron and ameliorates PD-like behavioral impairments. Thus, these results demonstrate for the first time that the neuroprotective effects of Lico.A are associated with microglia and anti-inflammatory effects in PD models.

## 1. Introduction

Parkinson’s disease (PD), an age-related neurodegenerative disorder, affects approximately 2% of elderly people over the age of 60. Its major clinical symptoms include resting tremor, rigidity and bradykinesia [1]. The prominent pathological feature of PD is the loss of dopaminergic neurons in the substantia nigra pars compacta (SNpc) region of the brain [2]. Although the etiology of PD remains elusive, recently evidence has suggested that chronic neuroinflammation caused by microglial activation may play an important role in the degenerative process. The first evidence that inflammatory processes are associated with PD came when McGeer et al. demonstrated the presence of activated microglia in the substantia nigra (SN) of post-mortem brain [3]. Since then, a large amount of evidence has demonstrated that neuroinflammation is closely related with the pathogenesis of several neurodegenerative disorders including PD [4,5].

As the resident macrophages of the brain, microglial cells play a crucial role in the innate immune response and are the first line of defense against microbial invasion and central nervous system (CNS) injury. Numerous reports indicate that uncontrolled over-activated microglia lead to neuroinflammation [6,7,8]. Over-activated microglial cells secrete various cytotoxic factors including nitric oxide (NO) and prostaglandin E2 (PGE2), and pro-inflammatory factors, such as tumor necrosis factor-α (TNF-α), interleukin-1β (IL-1β) and interleukin-6 (IL-6). Over the last two decades, studies in PD models have demonstrated that inflammation induced by lipopolysaccharide (LPS) can represent some characteristics of PD, including extensive activation of microglia and the selective loss of dopaminergic neurons in the nigrostriatal system [9,10,11].

LPS is an endotoxin found in the outer membrane of gram-negative bacteria and is a potent stimulator of both peripheral immune cells (macrophages and monocytes) and brain glia (microglia and astrocytes). It can up-regulate the release of various cytotoxic factors and pro-inflammatory cytokines. Thus, LPS is often used as a neuroinflammation elicitor to generate cellular/animal phenotypes of PD [12,13,14]. A large number of studies used co-culture of human SH-SY5Y cells with activated microglia induced by LPS as a cell culture model of PD to assess the neuroprotective effect of potential therapeutic agents [15,16,17]. Moreover, cultured primary mesencephalic neuron-glia cell are physiologically more relevant than immortalized cell lines to imitate PD model in vitro. The stereo-injection of LPS was used in a number of studies of PD animal model caused by neuroinflammation [18,19,20]. Thus, therapeutic intervention in inhibiting neuroinflammation which is incurred by LPS-activated microglia, would be an effective therapy in PD.

Epidemiological studies have demonstrated that the incidence of idiopathic PD is relatively low in chronic users of anti-inflammatory drugs [21,22]. Given the potential role of neuroinflammation in the pathogenesis of PD, drug candidates with anti-inflammatory properties are in high demand [23]. Licochalcone A (Lico.A) is one of the flavonoids compounds isolated from the roots of licorice [24]. It has also been reported that Lico.A exerts multiple bioactivities, including anti-inflammatory [25], anti-parasitic, [26] antioxidant [27] and anticancer [28,29,30]. Many studies have revealed that Lico.A has anti-inflammatory effects in peripheral tissues, such as lung, kidney and macrophages [31,32,33].

Given that neuroinflammation can cause dopaminergic neurodegeneration in PD, we investigated whether Lico.A inhibits microglial over-activation and alleviates dopaminergic neurodegeneration in PD models in vivo and in vitro. We assessed the specific neuroprotective mechanism of Lico.A in microglia. Our data show that Lico.A prevents the loss of dopaminergic neurons by suppressing microglial activation in PD models by inhibiting the ERK1/2 and NF-κB p65 pathways. Moreover, these findings provide additional potential targets for the therapeutic intervention of PD.

## 2. Results

### 2.1. Lico.A Inhibits NO and PGE2 Production by Suppressing iNOS and COX-2 Expression in LPS-Stimulated Microglial BV-2 Cells

**Figure 1:**
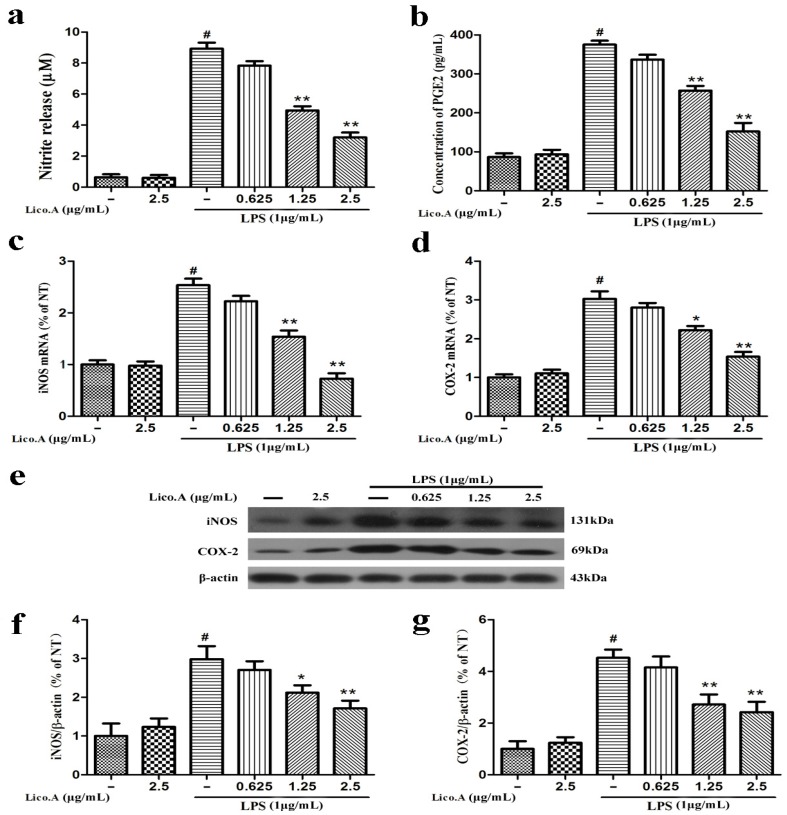
Licochalcone A (Lico.A) inhibits the production of nitric oxide (NO) and prostaglandin E2 (PGE2) and expression of iNOS and COX-2 in lipopolysaccharide (LPS)-stimulated BV-2 cells. (**a**) NO production was measured by Griess reagents; (**b**) The production of PGE2 in supernatant was analyzed by ELISA; (**c**,**d**) The mRNA levels of iNOS and COX-2 were measured by RT-PCR; (**f**,**g**) The protein levels of iNOS and COX-2 were analyzed by Western blot. All the experiments were repeated at least three times and similar results were observed. Values are mean ± SE (standard error), (*n* = 5 samples/group). ^#^ *p* < 0.01 vs. NT group (cultured in medium alone); ** *p* < 0.01, * *p* < 0.05 vs. LPS group.

The production of NO and PGE2 and the expression of iNOS and COX-2 are involved in LPS-activated neuroinflammation [34]. To explore whether Lico.A inhibits the production of NO and PGE2 and the expression of iNOS and COX-2, BV-2 cells were pretreated with Lico.A (0.625–2.5 µg/mL) for 1 h and then stimulated with LPS (1 µg/mL) for 24 h. The results indicated that BV-2 microglial cells treated with LPS for 24 h displayed a marked increase in nitrite (a stable oxidized product of NO) and PGE2 production by approximately nine and four folds, respectively. In addition, this nitrite and PGE2 production was dose-dependently inhibited by Lico.A pretreatment (Figure 1a,b). Lico.A alone did not have an effect on the production of NO and PGE2 in BV-2 cells. It has been shown that the production of NO and PGE2 in inflammatory cells is regulated by iNOS and COX-2, respectively [35]. To investigate the effect of Lico.A on the production of iNOS and COX-2, the BV-2 cells were pretreated with Lico.A (0.625–2.5 µg/mL) for 1 h and then stimulated with LPS (1 µg/mL) for 6 h. RT-PCR analysis revealed that LPS treatment observably increased the production of iNOS and COX-2 mRNA in BV-2 cells. In addition, the production of iNOS and COX-2 mRNA was also markedly attenuated by Lico.A (Figure 1c,d). Western blot analysis also showed that Lico.A significantly attenuated the protein expression of iNOS and COX-2 in LPS-stimulated BV-2 cells in a dose-dependently manner (Figure 1f,g). To examine the potential cytotoxicity of Lico.A, BV-2 cells were stimulated with different doses of Lico.A for 24 h, and cell viability was then examined by 3-(3,4-dimethylthiazole-2-yl)-2,5-diphenyl-tetrazoliumbromide (MTT) assay. The MTT assay results excluded non-specific cytotoxicity of Lico.A (data not shown), illustrating that Lico.A at non-cytotoxic levels suppresses LPS-induced inflammatory responses in BV-2 cells.

### 2.2. Lico.A Inhibits the Production of TNF-α, IL-6 and IL-1β and Down-Regulates the Expression of Cytokine mRNA in LPS-Stimulated BV-2 Cells

**Figure 2:**
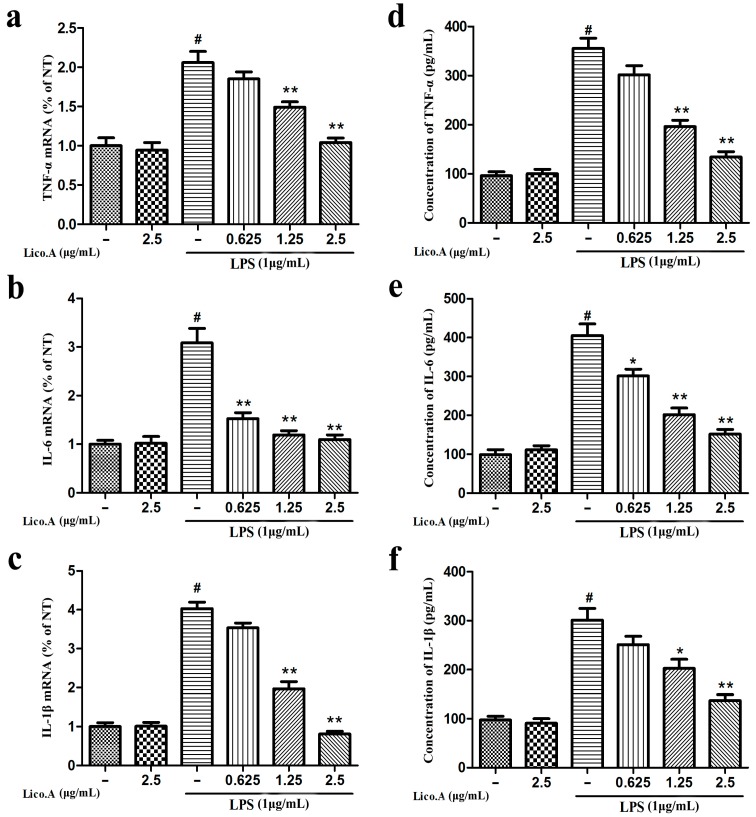
Lico.A inhibits the production of tumor necrosis factor-α (TNF-α), interleukin-1β (IL-1β) and interleukin-6 (IL-6) in LPS-stimulated BV-2 cells. (**a**–**c**) The mRNAs levels of TNF-α, IL-6 and IL-1β were examined by RT-PCR. (**d**–**f**) Extracellular levels of TNF-α, IL-6 and IL-1β in culture media were measured with commercial ELISA kits. All the experiments were repeated at least three times and similar results were observed. Values are mean ± SE, (*n* = 5 samples/group). ^#^ *p* < 0.01 vs. NT group; * p < 0.05, ** p < 0.01 vs. LPS group.

TNF-α, IL-6 and IL-1β secreted from microglia play a vital role in the inflammatory reaction in PD [36]. Therefore**,** to investigate whether Lico.A inhibits the production of pro-inflammatory cytokines (TNF-α, IL-6 and IL-1β), BV-2 cells were incubated with Lico.A (0.625–2.5 µg/mL) for 1 h and then stimulated with LPS (1 µg/mL) for 6 h. The RT-PCR analysis of BV-2 cells stimulated with LPS showed that Lico.A pretreatment inhibits the release of these pro-inflammatory cytokines in a dose-dependent manner (Figure 2a–c). Furthermore, ELISA analysis revealed that the production of these pro-inflammatory mediators induced by LPS for 24 h was dramatically inhibited by Lico.A pretreatment for 1 h (Figure 2d–f). These results indicate that Lico.A down-regulates the expression of these cytokines at the transcriptional level.

### 2.3. Lico.A Suppresses the Phosphorylation of the ERK 1/2 MAPK Signaling Pathway in LPS-Induced BV-2 Cells

**Figure 3:**
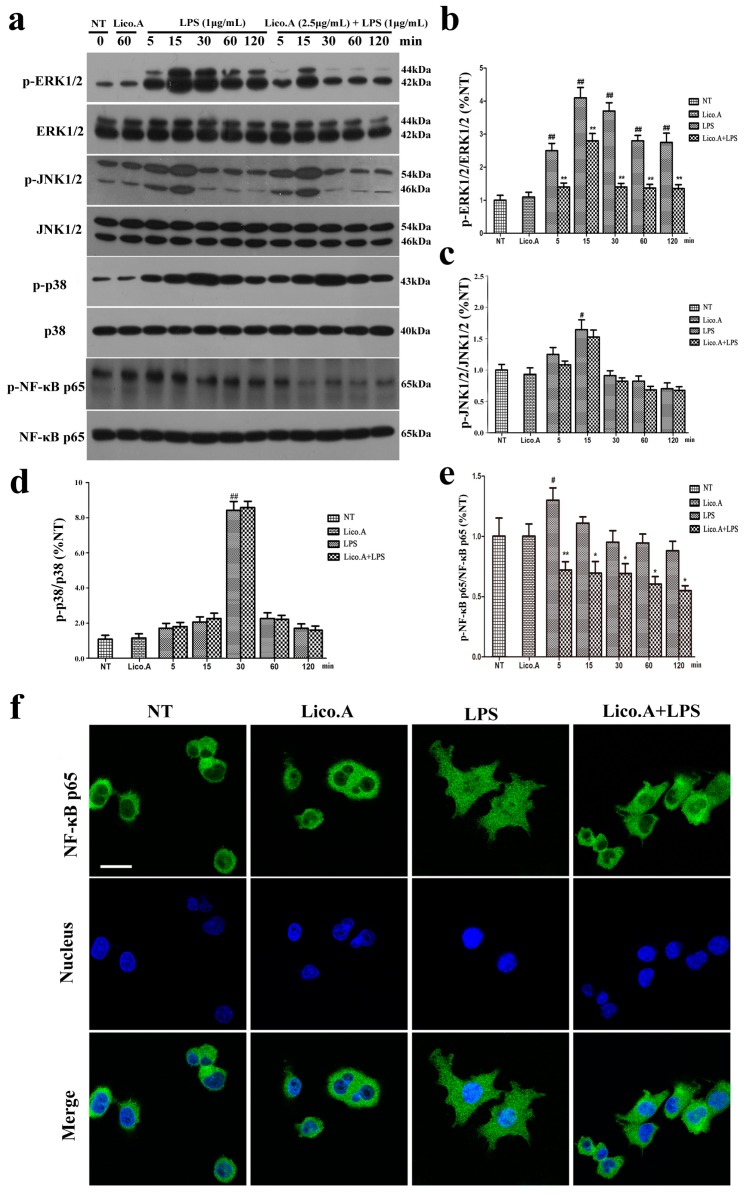
Lico.A suppresses extracellular signal-regulated kinase (ERK1/2) and nuclear factor κB (NF-κB) activation in LPS-stimulated BV-2 cells. (**a**–**e**) The phosphorylation of ERK1/2, JNK1/2, p38 and NF-κB p65 was analyzed by Western blot. A representative immunoblot is shown. All the experiments were repeated at least three times and similar results were observed; (**f**) NF-κB p65 nuclear translocation was inspected by ICC-IF assay. Representative photomicrographs of NF-κB p65 subunit are shown. Scale bars, 50 µm. Values are mean ± SE, (*n* = 5 samples/group). ^#^ *p* < 0.01, ^##^ *p* < 0.001 vs. NT group; * *p* < 0.05, ** *p* < 0.01 vs. LPS group.

We explored the effect of Lico.A on the mitogen-activated protein kinases (MAPKs) signaling pathway, which plays an important role in the regulation of inflammatory responses [37]. The BV-2 cells were pretreated with Lico.A (2.5 µg/mL) for 60 min and then stimulated with LPS for different periods (0, 5, 15, 30, 60, or 120 min). The results of the Western blot showed that LPS (1 μg/mL) markedly increased the phosphorylation of p38, ERK1/2, and JNK1/2 in a time-dependent manner (Figure 3a). Lico.A pretreatment of the BV-2 cells considerably attenuated the LPS-induced phosphorylation of ERK1/2 (Figure 3b), while no effect was observed on the phosphorylation of JNK1/2 or p38 (Figure 3c,d).

### 2.4. Lico.A Attenuates the NF-κB p65 Inflammatory Signaling Pathway in LPS-Activated BV-2 Cells

The activated NF-κB signaling pathway contributes to the production of pro-inflammatory enzymes and pro-inflammatory cytokines in microglia [38]. To investigate how Lico.A inhibits the expression of enzymes and cytokines, the effect of Lico.A on the phosphorylation of NF-κB p65 and the translocation of NF-κB p65 in BV-2 cells was examined by Western blot and immunocytochemistry, respectively. The BV-2 cells were pretreated with Lico.A for 60 min and then stimulated with LPS for different periods (0, 5, 15, 30, 60, or 120 min). The Western blot analysis showed that the phosphorylation of NF-κB p65 was inhibited by Lico.A (Figure 3e). After stimulating BV-2 cells with LPS for 60 min, the translocation of the NF-κB p65 subunit into the nucleus was examined by an immunocytochemistry-immunofluorescence (ICC-IF) assay, which showed that the translocation was inhibited by Lico.A (Figure 3f). Together, these results indicate that, through the inhibition of NF-κB activation, Lico.A is also likely to suppress the production of NO, PGE2, iNOS, COX-2 and pro-inflammatory factors in LPS-activated BV-2 cells.

### 2.5. Lico.A Prevents the Degeneration of Dopaminergic Neurons in LPS-Induced PD Models In Vitro

**Figure 4:**
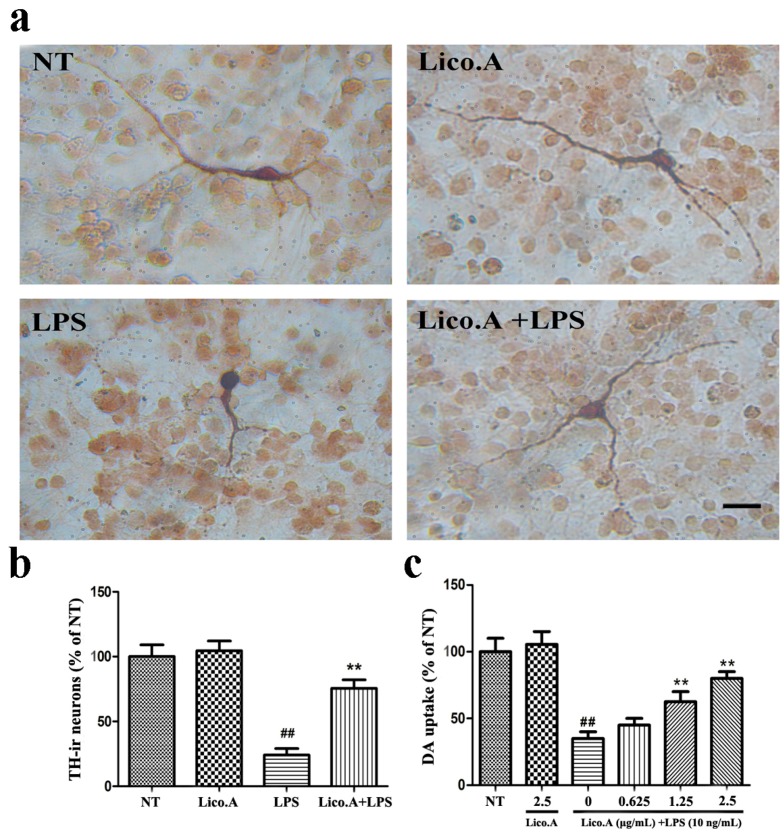
Lico.A prevents LPS-induced degeneration of dopaminergic neurons in cultured mesencephalic neuron-glia. (**a**) The degeneration of dopaminergic neurons was assessed by TH immunostaining. Scale bar, 250 µm; (**b**) The TH-ir neuron was counted; (**c**) The level of [^3^H]DA uptake was measured. All the experiments were repeated at least three times and similar results were observed. Values are mean ± SE, (*n* = 5 samples/group). ^#^ *p* < 0.01, vs. NT group and * *p* < 0.05, ** *p* < 0.01 vs. LPS group.

In this study, we established an in vitro PD model by culturing primary mesencephalic neuron-glia from the embryonic rat ventral midbrain. To investigate whether the LPS-induced damage of dopaminergic neurons could be prevented by Lico.A, cultured mesencephalic neuron-glia were pretreated for 60 min with Lico.A before being treated with 10 ng/mL LPS. Seven days later, the degeneration of dopaminergic neurons was assessed by tyrosine hydroxylase (TH) immunostaining and [^3^H]DA uptake. Morphologically, the remaining tyrosine hydroxylase-immunoreactive (TH-ir) neurons in the LPS-treated cultures had fewer dendrites and shorter or evenly truncated axons (Figure 4a). The LPS treatment reduced the number of TH-ir neurons by 80%, compared with the no treatment (NT) group (Figure 4b). However, Lico.A pretreatment dramatically inhibited the reduction of the number of TH-ir neurons, suggesting that Lico.A markedly attenuates the LPS-induced loss of TH-ir neurons. The [^3^H]DA uptake assays illustrated that the LPS treatment reduced the uptake capacity by approximately 60%, compared with the NT group, and the LPS-induced reduction in [^3^H]DA was abated by Lico.A pretreatment in a concentration dependent manner (Figure 4c). Through the TH immunostaining and [^3^H]DA uptake, the neuron-glia cells treated with 2.5 µg/mL Lico.A in the cultures were similar to those in the NT cultures, suggesting that Lico.A has no obvious toxicity.

### 2.6. Lico.A Treatment Ameliorates the Behavioral Dysfunction of LPS-Induced PD Model Rats

**Figure 5:**
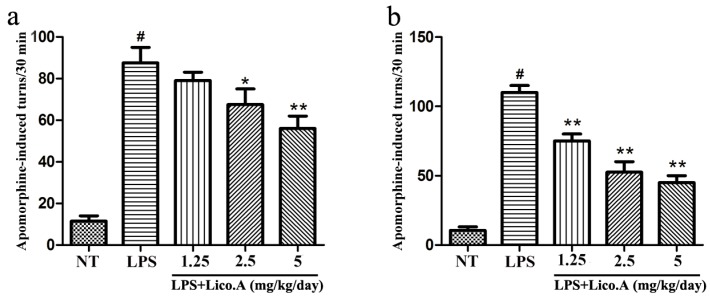
Lico.A ameliorates the behavioral dysfunction in LPS-induced PD model rats. (**a**) The number of turns two weeks after apomorphine treatment was counted (*n* = 18 per group); (**b**) The number of turns four weeks after apomorphine treatment was calculated (*n* = 18 per group). The experiments were repeated three times. Values are mean ± SE. ^#^ *p* < 0.01, vs. NT group and * *p* < 0.05, ** *p* < 0.01 vs. LPS group.

To examine the effect of Lico.A treatment on motor dysfunction, LPS-induced PD model rats were subjected to behavioral tests two and four weeks after LPS injection. Apomorphine treatment leads to rotational behavior towards the injection side, which is a frequently-used method for detecting damage of the dopaminergic system [39]. Rats were randomly grouped and then pretreated with Lico.A (1.25, 2.5, or 5 mg/kg/ay) or vehicle three days before LPS injection. Subsequently, they were continuously injected with Lico.A or vehicle for 25 days (28 days in total). Our findings revealed that behavioral dysfunction was quite obvious two weeks after LPS lesion (Figure 5a). Furthermore, we found that Lico.A treatment significantly reduced apomorphine-induced rotation after LPS lesion for four weeks (Figure 5b). The results indicate that Lico.A treatment improves the motor dysfunction of LPS-induced PD model rats. 

### 2.7. Lico.A Treatment Prevents the Loss of TH-Positive Cells in the SN of LPS-Induced PD Model Rats

**Figure 6:**
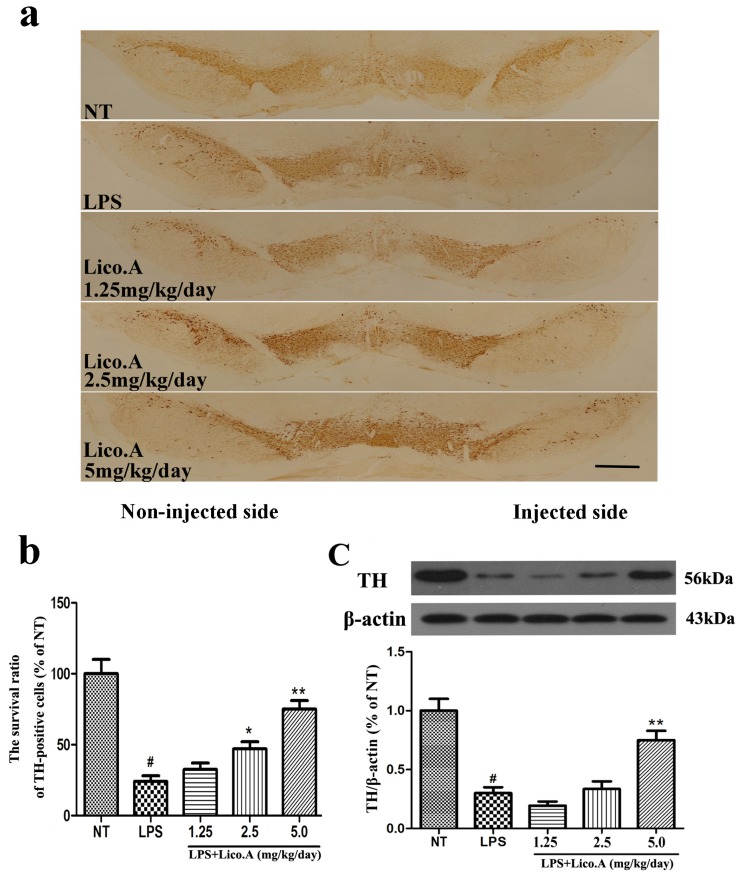
Lico.A treatment increases the number of TH-positive cells and TH expression in the SN of LPS-induced PD model rats. (**a**) Staining of TH-positive neurons in the SN was processed (*n* = 6 per group). Scale bar, 1.0 mm; (**b**) The survival ratio of the dopaminergic neurons in the SNpc (TH-positive cells on the injected side versus the TH-positive cells on non-injected side) was calculated; (**c**) TH expression in the SN was assayed by Western blot (*n* = 6 per group). A representative immunoblot is shown. The experiments were repeated three times. Values are mean ± SE. ^#^ *p* < 0.01, vs. NT group and * *p* < 0.05, ** *p* < 0.01 vs. LPS group.

The unilateral intranigral injection of LPS is a model of studying the selective effects of inflammatory reactions on the dopaminergic system [40,41]. TH, the rate-limiting enzyme in the synthesis of catecholamines, plays a vital role in DA synthesis [42]. In this study, PBS or 10 μg of LPS was unilaterally injected into the right SN of rats. The animals were sacrificed after 4 weeks of LPS treatment. To further investigate the protective effect of Lico.A on dopaminergic neurons, immunohistological analysis of TH expression was carried out in the LPS-induced PD model in vivo. The results demonstrated that the number of TH-positive neurons on the side of injection was similar to that on the contralateral side in the NT group (Figure 6a). The animals that received the vehicle treatment after LPS intranigral injection showed marked loss of TH-ir neurons and a considerable decrease in dendrites in the SN of LPS-injected rats (*p* < 0.01). However, Lico.A treatment (1.25, 2.5 and 5 mg/kg/day) dramatically inhibited these changes (Figure 6a,b). The effects of Lico.A on TH protein expression in the SN were examined by Western blot analysis. The results demonstrated an obvious decrease in TH expression in the SN of the LPS-induced PD model rats (Figure 6c, *p* < 0.01). The Lico.A treatment suppressed the damage to TH, which was induced by LPS in a concentration-dependent manner, indicating that it can prevent dopaminergic neurodegeneration. 

### 2.8. Lico.A Treatment Inhibits Microglial Activation and Down-Regulates mRNA Expression of Neuronal Toxic Factors in the SN of LPS-Induced PD Model Rats

**Figure 7:**
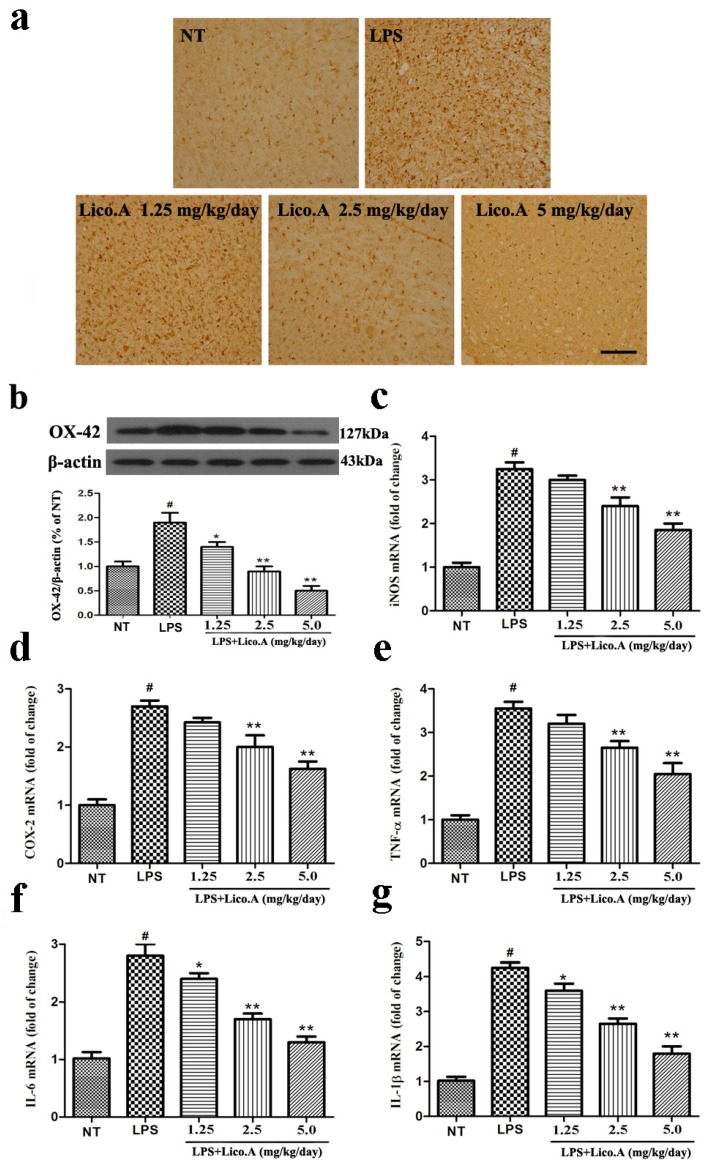
Lico.A treatment inhibits microglial activation and down-regulates mRNA expression of neuronal toxic factors in the SN of LPS-induced PD model rats. (**a**) The morphological changes of the microglia in the SN were shown via Iba-1 immunohistochemical staining (*n* = 6 per group). Representative photomicrographs of the SN area are shown. Scale bar, 100 μm; (**b**) The expression of OX-42 was assayed by Western blot (*n* = 6 per group). A representative immunoblot is shown; (**c**–**g**) The expression of iNOS, COX-2, TNF-α, IL-6 and IL-1β mRNA in the SN of LPS induced PD model rats was analyzed by Real-time RT-PCR (*n* = 6 per group). All the experiments were repeated at least three times and similar results were observed. Values are mean ± SE. ^#^ *p* < 0.01, vs. NT group and * *p* < 0.05, ** *p* < 0.01 vs. LPS group.

It has been reported that microglial activation is associated with the loss of dopaminergic neurons in LPS-induced PD animal models [43]. Consistent with other reports, microglia transform from resting cells to activated large cells after LPS injection [44]. Therefore, we assessed whether the inhibition of LPS-induced microglial activation was involved in the neuroprotective effect of Lico.A. As a specific marker for microglial activation, the expression of ionized calcium binding adaptor molecule-1 (Iba-1) was examined by immunohistochemical staining. The activation of microglia is dramatically suppressed by Lico.A treatment in a dose-dependent manner (Figure 7a). To obtain quantitative data, the SN of the rats was dissected. Microglial activation was inspected by Western blot analysis with an OX-42 antibody. The results confirmed that Lico.A treatment suppressed LPS-induced microglial activation in a concentration-dependent manner (Figure 7b). Since activated-microglia are the main source of neuronal toxic factors in the brain, and the present study showed that Lico.A inhibited microglial activation, we hypothesized that Lico.A treatment could inhibit the LPS-induced production of neuronal toxic factors. Thus, we measured the mRNA expression of iNOS, COX-2, TNF-α, IL-1β and IL-6 in the SN. The results showed that LPS injection significantly up-regulated the expression of iNOS, COX-2, TNF-α, IL-1β and IL-6 mRNA, but the Lico.A treatment down-regulated the expression in a concentration-dependent manner (Figure 7c–g). The results suggest that Lico.A treatment suppresses the neuroinflammatory reaction induced by microglial activation in vivo.

## 3. Discussion

Inflammatory processes in the CNS are believed to play an important role in neurodegenerative diseases caused by neuronal cell death [45]. Chronic neurodegeneration is accompanied by an inflammatory response, which is induced by the activation of microglial cells in the CNS. Many studies suggest that microglia activation plays a pivotal role in the process of neuroinflammation [46]. Over-activated microglia could secrete a variety of cytotoxic factors, such as NO, PGE2, TNF-α, IL-1β and IL-6 [47,48,49]. These factors contribute to the inflammatory reaction in PD. It is widely recognized that multiple immunological stimuli such as α-synuclein, TNF-α and LPS can trigger microglial activation, leading to sustained chronic inflammation in the dopaminergic neurodegenerative process [10,20]. Many PD animal models are induced by various pathways and have been widely used by researchers [50]. For instance, 6-hydroxydopamine (6-OHDA) is used to establish a PD model through oxidative stress, 1-methyl-4-phenyl-1,2,3,6-tetrahydropyridine (MPTP) and rotenone through mitochondrial complex I inhibition, and LPS is used to establish a PD model through its glial cell activation. The PD models induced by these toxins have been widely used to test the effects of neuroprotective agents.

It has been proven that LPS could kill dopaminergic neurons through glial cell activation in in vitro cell experiments, which is accompanied by an increased release of cytokines [51,52]. A cultured primary ventral mesencephalon (VM) of an embryonic rat was prepared to mimic the PD model in vitro. The unilateral stereotaxic injection of LPS into the rat’s substantia nigra also results in the loss of neurons and the destruction of the nigro-striatal pathway. After damage of the pathway, the rat is treated with amphetamine or apomorphine, which leads to its asymmetric motor function [40,41]. In the in vivo PD model, the injection of LPS (10 µg in 2 µL of PBS for 10 min) into the substantia nigra leads to the activation of microglia, the loss of dopaminergic neurons and consequent impairments in spontaneous motor function [53]. Therefore, it can be concluded that the LPS-induced PD model is effective in studying the role of neuroinflammation in the process of dopaminergic neurodegeneration. Additionally, this model can be used to explore new therapy agents.

Lico.A has been proven to have multiple potent bioactivities, including anti-inflammatory and anti-osteoporosis activities [54]. However, the neuroprotective effect of Lico.A remains unclear. It has been shown that several different signaling pathways were inhibited by Lico.A, such as the p38 MAPK pathway in LPS-stimulated RAW264.7 cells, the Stat3 pathway in IL-3 treated Ba/F3/MSCV cells and the mTOR pathway in SiHa cells [55,56,57]. These findings raise the question of whether Lico.A affects different in different pathways. Here, we tested the mechanisms of Lico.A in LPS-stimulated BV-2 cells. We found that the Lico.A pretreatment suppressed neuroinflammatory reactions by blocking the ERK1/2 and NF-κB p65 pathways in microglia. Thus, Lico.A may inhibit the death of dopaminergic neurons that is caused by neuroinflammation in the pathological process of PD.

NO, an important regulatory mediator in cell survival and death, plays a very important pro-inflammatory role during various physiological and pathological processes [58]. Excessive production and accumulation of NO caused by activated microglial cells is believed to lead to neuronal death during ischemia, trauma and neurodegenerative diseases in vivo and in vitro [59,60,61]. Studies have demonstrated that the inhibitors of NO have significant neuroprotective effects during pathological processes. PGE2 is a bioactive lipid that exerts diverse effects on immune surveillance, cell proliferation, inflammation and apoptosis [62]. PGE2 plays a critical role in regulating various aspects of the inflammatory response. The role of PGE2 in inflammation has been confirmed. The biosynthesis of NO and PGE2 is regulated by iNOS and COX-2 molecules, respectively [63]. The up-regulation of iNOS and COX-2 contributes to the development of many chronic inflammatory diseases, and it has been reported that the inhibitors of iNOS and COX-2 can block microglial activation and play a role in neuroprotection. In our study, Lico.A suppressed not only NO and PGE2 production but also iNOS and COX-2 expression in LPS-activated BV-2 microglial cells. TNF-α, IL-1β and IL-6 are three main pro-inflammatory cytokines produced by activated microglia during CNS inflammation. TNF-α plays a central role in initiating and regulating the cytokine cascade during an inflammatory response [64]. IL-1β promotes the inflammatory reaction of glial cell and leads to diseases related to neuronal loss, such as ischemic and traumatic brain injury and neurodegenerative disease [65]. In addition, IL-1β produced by LPS-activated microglia can activate the tumor suppressor p53 to induce apoptosis of neural precursor cells [66]. IL-6 is a multifunctional cytokine that plays an important role in host defense and in regulating the inflammatory response [67]. The results of the present study indicated that Lico.A significantly inhibited LPS-induced TNF-α, IL-1β and IL-6 production. The neuroprotective effect of Lico.A was further confirmed by measuring the expression of pro-inflammatory genes in brain tissues, where Lico.A could suppress the upregulation of mRNA of neuronal toxic factors in LPS-induced PD rats. 

After microglia are treated with LPS, multiple signaling pathways are activated. It has been shown that the activation of the MAPKs and NF-κB signaling pathways is involved in the regulation of inflammatory mediators in microglia [68]. Thus, the effects of Lico.A on MAPKs (p38, ERK, and JNK) and NF-κB p65 were investigated in this study. It is widely -accepted that the LPS treatment of BV-2 cells can activate all the MAPKs and NF-κB p65 pathways. We found that the phosphorylation of ERK1/2 and NF-κB p65, which was induced by LPS, was suppressed by Lico.A treatment. However, Lico.A has no effect on the phosphorylation of p38 and JNK1/2. Previous studies have shown that LPS stimulation leads to the translocation of the NF-κB p65 subunit to the nucleus in BV-2 cells. It was illustrated that after Lico.A pretreatment of LPS-stimulated BV-2 cells, NF-κB p65 nuclear translocation was diminished. Many studies have suggested that the selective inhibition of the activation of the MAPKs and NF-κB pathways in microglia can exert neuroprotective effects in PD rat models in vivo and in vitro [69,70]. Furthermore, previous immunohistochemical reports demonstrated NF-κB activation in dopaminergic neurons among PD patients [71]. Therefore, Lico.A may exert neuroprotective effects by inhibiting MAPKs and NF-κB in microglial cells.

It has been reported that in cultured mesencephalic neuron-glia, LPS can induce microglial activation, and the activated microglia have been shown to release cytotoxic factors such as NO, TNF-α and IL-1β, which lead to the consequent degeneration of dopaminergic neurons [72]. Moreover, the cultured neuron–glia is made up of approximately 11% microglia, 50% astrocytes and 39% neurons. However, it has been revealed that LPS fails to induce astrocytic activation in cultured primary glia once microglia are depleted by leucine-methyl esther [73]. A recent report has confirmed that activation of astrocytic induced by LPS does not produce strong toxicity to either dopaminergic or non-dopaminergic neurons [74]. This is because LPS-stimulated astrocytes produce more neurotrophic factors that can antagonize the increased pro-inflammatory mediators. Thus, we assessed the degeneration of dopaminergic neurons via TH immunostaining and [^3^H]DA uptake in cultured mesencephalic neuron-glia. Morphologically, the remaining TH-ir neurons had dramatically fewer dendrites and shorter or evenly truncated axons in the LPS-treated cultures. The [^3^H]DA uptake assays demonstrated that the reduction of [^3^H]DA induced by LPS was abated by Lico.A pretreatment in a concentration-dependent manner. Collectively, these results provide indirect evidence that Lico.A can protect dopaminergic neurons from LPS neurotoxicity and exert its neuroprotective effects by inhibiting microglial activation in cultured mesencephalic neuron-glia.

LPS injection to the SN of rats leads to microglial over-activation, which selectively produces lasting degeneration of dopaminergic neurons. It has been reported that LPS injection to the SN of rats can mimic the pathological and clinical features of PD [53]. These PD models have been widely used in drug discovery, and a variety of agents, such as Triptolide and Tiagabine, have been evaluated for their potential neuroprotective effects in LPS-induced PD models [17,75]. Furthermore, there is no detectable damage to either GABAergic or serotoninergic neurons in the rat’s nigra after LPS injection, indicating that LPS selectively induces the loss of dopaminergic neurons in the nigrostriatal system [41]. A number of studies have confirmed these results and the increased levels of pro-inflammatory mediators, including IL-1β, TNF-α, IL-6 and NO in LPS-induced PD models [76,77]. Therefore, PD models serve as powerful tools for the study of pathological mechanisms and the identification of potential therapeutic agents. We investigated the motor dysfunction of these PD model rats with rotational behavior tests. Apomorphine-induced rotation is often used to evaluate the degree of damage to the dopaminergic system. It was found that the number of apomorphine-induced rotation significantly increased in LPS-induced PD model rats, but Lico.A treatment showed a therapeutic effect on this behavioral dysfunction. Further studies demonstrated that Lico.A could inhibit the over-activation of microglia, the release of pro-inflammatory factors and the damage of dopaminergic neuronal damage. These data showed that Lico.A played a neuroprotective role in the LPS-induced PD model in vitro.

In summary, our study has revealed that Lico.A treatment prevents dopaminergic neurodegeneration by inhibiting microglia-mediated neuroinflammation. Several lines of evidence presented in this study demonstrated that Lico.A exerted potent neuroprotection on dopaminergic neurons against LPS-induced neurotoxicity in PD models in vivo and in vitro. The underlying mechanism is that Lico.A inhibites LPS-induced microglial activation via downregulation the activation of ERK1/2 and NF-κB p65 pathways. Taken together, these results suggest that Lico.A possesses a notable neuroprotective property, indicating its potential as a drug candidate for PD.

## 4. Materials and Methods

### 4.1. Reagents

Lico.A, a pale yellow powder with 99% purity, was purchased from Pufei De Biotech (Chengdu, China). The Trizol reagent, Poly-L-lysine (PLL), Apomorphine, Griess reagent, [^3^H]DA in Krebs–Ringer buffer, LPS from *Escherichia coli*, O55:B55, and Dimethyl sulfoxide (DMSO) were obtained from Sigma-Aldrich (St. Louis, MO, USA). The 0.25% trypsin and Penicillin–streptomycin (PS) were purchased from Invitrogen (Carlsbad, CA, USA). The Dulbecco’s modified Eagle’s medium (DMEM), minimum essential medium (MEM), fetal bovine serum (FBS) and horse serum (HS) were purchased from Gibco (Grand Island, NY, USA). The PrimeScript^®^ 1st Strand cDNA Synthesis Kit was purchased from Takara Biotechnology (Dalian, China). The SYBR Green QuantiTect RT-PCR Kit was purchased from Roche (South San Francisco, CA, USA). The PGE2, TNF-α, IL-6 and IL-1β ELISA kits were purchased from R&D Systems (Abingdon, UK). 

### 4.2. Cell Culture and Treatment

The murine microglia cell line, BV-2 cells, was obtained from the Cell Culture Center at the Institute of Basic Medical Sciences, Chinese Academy of Medical Sciences (Beijing, China). The cells were similar to primary microglia in producing NO, PGE2 and various cytokines after stimulation. Cells were cultured in DMEM supplemented with 10% FBS and 50 U/mL penicillin-50 μg/mL streptomycin at 37 °C in a humidified cell incubator under a 95%/5% (*v*/*v*) mixture of air and CO2. The culture medium was changed once every two days, and the BV-2 cells were treated with the method of trypsinization (0.05%, *w*/*v*) at approximately 80% confluence. To reduce mitogenic effects, the cells used in the experiments were washed once with DMEM medium and pre-cultured in serum-free DMEM medium for at least 4 h prior to treatments. The BV-2 cells were pretreated with various concentrations of Lico.A (dissolved in DMSO) for 1 h and then stimulated with LPS.

### 4.3. Animals and Treatment

All experiments were done in accordance with approved animal protocols and guidelines established by the Institutional Animal Care and Use Committee of Jilin University (approved on 27 February 2015, Protocol No. 2015047). Efforts were made to minimize animal suffering and to reduce the number of animals used. Nine- to eleven-week-old female Wister rats (290 to 320 g) were obtained from the Center of Experimental Animals of the Baiqiuen Medical College of Jilin University. The rats were randomly divided into groups (*n* = 18 in each group) and supplied with food and water in plastic cages under conventional conditions at 22 °C, 50–60% humidity and a 12 h light/12 h dark cycle. The rats were anaesthetized with sodium pentobarbital (45 mg/kg, i.p.) and secured in a stereotaxic apparatus (David Kopf Instruments, Tujunga, CA, USA). The right SNpc was given the injection of LPS (10 µg dissolved in PBS with total volume of 2 µL) or PBS at a rate of 0.2 µL/min (anteroposterior (AP) −5.2 mm, lateral (LAT) 2.1 mm and dorsoventral (DV) 7.8 mm). Then, the needle was left in situ for 10 min to avoid reflux along the injection track. The rats’ intraperitoneal injection of Lico.A (1.25, 2.5 and 5 mg/kg, dissolved in 5 µL of DMSO and then diluted with 1 mL of PBS, once daily) lasted for 3 days prior to surgery. The rats of the NT group received an equal volume of the vehicle solution. After the surgery, the animals were given Lico.A for 25 days. After the last behavioral test, the rats (*n* = 6 in each group) were transcardially perfused with paraformaldehyde for immunohistochemical analysis of TH (1:1000; Abcam, Cambridge, CA, USA) and Iba-1 (1:200, Proteintech, Chicago, IL, USA). The remaining brains of the rats (*n* = 12) were rapidly removed. Then, fresh SN tissues were isolated for the measurement of inflammatory mediators, TH and the other biomarker OX-42.

### 4.4. NO Assay

Nitrite, a soluble oxidation product of NO, was assayed in the culture media with the Griess reagent according to the manufacturer’s instructions. Briefly, BV-2 cells (5 × 10^5^ cells/mL) were seeded in the 24-well plates and then treated with Lico.A and/or LPS for 24 h. The supernatant (50 μL) was mixed with an equal volume of Griess reagent (parts 1 and 2) in a 96-well plate and incubated in the dark for 20 min at room temperature. Finally, the absorbance values were read at a wavelength of 540 nm by a microplate reader (BioTek, Winooski, VT, USA) and nitrite concentrations were based on the standard curve of sodium nitrite.

### 4.5. Enzyme-Linked Immunosorbent Assay (ELISA)

The BV-2 cells were plated into 24-well plates (2 × 10^5^ cells/well). The cells were pretreated with Lico.A for 1 h and then stimulated with LPS (1 μg/mL) for 24 h. The media was collected and centrifuged after treatment. The levels of PGE2, TNF-α, IL-6 and IL-1β were measured with ELISA kits according to the manufacturer’s protocol.

### 4.6. RNA Isolation and Quantitative PCR

The total RNA of BV-2 cells and SN tissues were extracted with the Trizol reagent using the supplier’s protocol. After the concentration of RNA was measured at 260 and 280 nm of absorbance by a spectrophotometer, 2 μg of RNA was reverse-transcribed (RT) into cDNA with the PrimeScript^®^ 1st Strand cDNA Synthesis Kit according to the manufacturer’s instructions. The real-time PCR was then analyzed with SYBR Green QuantiTect RT-PCR Kit, and each sample was independently analyzed three times. The PCR amplifications were performed with 40 cycles of denaturation at 95 °C for 10 s, annealing at 60 °C for 30 s and extension at 72 °C for 30 s. The relative levels of gene expression of each mRNA were calculated by normalization to β-actin mRNA expression according to the 2^-ΔΔ*C*t^ method. The primer sequences for the tested genes are shown in Table 1 and Table 2.

### 4.7. Western Blotting Analysis

After the last behavioral test, the SN was rapidly dissected, frozen, and stored in a deep freezer at −80 °C until further assayed. The Western blot assay was performed according to standard protocols. The SN and BV-2 cells were lysed in RIPA lysis buffer (Beyotime Inst. Biotech, Beijing, China) containing phenyl-methylsulfonyol fluoride (PMSF). After 30 min, these lysates were obtained by centrifugation at 12,000 rpm for 10 min at 4 °C. The protein concentration of the samples was then examined with a bicinchoninic acid protein assay kit (Beyotime Inst. Biotech, Beijing, China) with bovine serum albumin as a standard. After the extracted lysates were mixed in loading buffer and then boiled for 5 min, equivalent amounts of protein (50 µg) were loaded and run on a single track of a 12% or 10% SDS-polyacrylamide gel and then transferred to polyvinylidene difluoride membranes (PVDF: Millipore, Bedford, MA, USA). The membranes were blocked with 5% skim milk-TBST for 2 h at room temperature and then incubated with primary antibodies against iNOS (1:1000), COX-2 (1:1000), OX-42 (1:1000), TH (1:1000) (Abcam, Cambridge, UK); ERK1/2 (1:2000), JNK1/2 (1:2000), p38 (1:2000), phospho-ERK1/2 (1:10,000), phospho-JNK1/2 (1:1000), phospho-p38 (1:1000), NF-κB p65 (1:10,000), phospho-NF-κB p65 (1:1000) (Cell Signaling Technology, MA, USA) and β-actin (1:10,000) (Santa Cruz, CA, USA) overnight at 4 °C. After being incubated, the blots were washed four times for 15 min each in TBST and then incubated with the secondary antibodies goat anti-rabbit (1:2000) or goat anti-mouse (1:2000) (Santa Cruz, CA, USA) for 1 h at room temperature. Next, the blots were again washed four times for 15 min each in TBST and then detected with enhanced chemiluminescence. The blots were developed with ECL Western blot Detection Reagents (Amersham Pharmacia Biotech, Tokyo, Japan).

### 4.8. Immunofluorescence Assay

The BV-2 cells were cultured onto poly-*L*-lysine-coated slips in 24-well plates and then treated with Lico.A (2.5 μg/mL) or LPS to detect the intracellular location of the NF-κB p65 subunit. Thus, the cells were fixed with Immunol Staining Fix Solution (Beyotime Inst. Biotech, Beijing, China) at room temperature for 10 min, washed three times with PBS and then permeabilized with PBS that contains 0.1% Triton X-100 for 10 min. The slips were washed three times with PBS again and then blocked in 5% normal goat serum at room temperature for 2 h. To analyze the location of the NF-κB p65 subunit, the cells were incubated overnight with the anti-NF-κB p65 antibody (1:1000 Cell Signaling Technology, MA, USA), subsequently washed three times with PBS and then incubated with secondary antibody for 1 h at room temperature. After being washed three times with PBS, cells nuclei were counter-stained with DAPI. Cover slips were then washed three times with PBS and mounted onto slides with fluorescent mounting medium. The representative images were obtained from 10 fields of view per treated group.

### 4.9. Rat Mesencephalic Neuron-Glia Cultures

The isolation of ventral mesencephalon (VM) neurons was carried out according to the method proposed by Bollimpelli VS [78]. The VM of Wistar rats was dissected from the fetal brain at embryonic day 14 (E14). In brief, VM tissues were cut into small segments and then dissociated as single cells in a mechano-enzymatic method, which involves a protease treatment with 2.5 mg/mL trypsin, 0.1 mg/mL DNAse type I (Sigma-Aldrich) and additional mechanical shearing. After centrifugation, cells were seeded at 1 × 10^5^ per well in 24-well culture plates which were pre-coated with poly-l-lysine (1 mg/mL). The cells were maintained in a maintenance medium consisting of MEM supplemented with 10% heat-inactivated FBS, 10% heat-inactivated HS, 2 mM L-glutamine, 1 mM sodium pyruvate, 100 μM nonessential amino acids and 50 U/mL penicillin-50 μg/mL streptomycin at 37 °C in a humidified atmosphere of 5% CO_2_ and 95% air. Each experiment was repeated three times. Seven-day-old cultures were used for Lico.A treatment.

### 4.10. TH and Iba-1 Immunohistological Analysis

The VM neurons and the rats’ brains were processed by Tolosa A and Ha SK, respectively for immunostaining analysis [79,80]. The rats’ dopaminergic neurons were detected with the anti-TH antibody. The microglia were treated with the anti-Iba-1 antibody. These experimental procedures were followed Li Y’s method [81]. To observe the number of TH positive cells, the cells on the injected side and non-injected side were respectively quantified by three researchers who were blinded to the experimental treatments. The ratios of these scores were determined.

### 4.11. Motor Function Test with the Rotarod

To examine the motor balance and coordination of the rats, we carried out an accelerating rotation test. In short, to acclimate to the rotarod apparatus, rats were placed into cylinders that were attached to a training session (10 rpm for 10 min) before treatment. Then, they were intraperitoneally injected with 5 mg/kg apomorphine. The measurements of motor functional activity began at 5 min after injection and lasted for 30 min under minimal external stimuli. The number of turns during the entire 30-min testing period was counted.

### 4.12. [^3^H]DA Uptake Assay

The mesencephalic neuron-glia were cultured for 20 min at 37 °C with 1 μM [^3^H]DA in Krebs–Ringer buffer (Sigma-Aldrich). After being washed three times with ice-cold Krebs–Ringer buffer, the cells were lysed in 1 N NaOH. The radioactivity was measured by a liquid scintillation counter (Tri-Carb, Packard, Meriden, CT, USA). The non-specific DA uptake observed was subtracted in the presence of mazindol (10 μM).

### 4.13. Statistical Analysis

All results are presented as mean ± SE, and were analyzed by SPSS 12.0 statistical software package. The differences between all data were assessed by one-way analysis of variance (ANOVA). A value of *p* < 0.05 was considered to be statistically significant.

## Figures and Tables

**Table 1:** Primers of mice for real-time RT-PCR.

Gene	Sequences	Length (bp)
*β-actin*	(F) 5′-GTCAGGTCATCACTATCGGCAAT-3′	147
	(R) 5′-AGAGGTCTTTACGGATGTCAACGT-3′	
*iNOS*	(F) 5′-GAACTGTAGCACAGCACAGGAAAT-3′	158
	(R) 5′-CGTACCGGATGAGCTGTGAAT-3′	
*COX-2*	(F) 5′-CAGTTTATGTTGTCTGTCCAGAGTTTC-3′	127
	(R) 5′-CCAGCACTTCACCCATCAGTT-3′	
*TNF-α*	(F) 5′-CCCCAAAGGGATGAGAAGTTC-3′	136
	(R) 5′-CCTCCACTTGGTGGTTTGCT-3′	
*IL-1β*	(F) 5′-GTTCCCATTAGACAACTGCACTACAG-3′	139
	(R) 5′-GTCGTTGCTTGGTTCTCCTTGTA-3′	
*IL-6*	(F) 5′-CCAGAAACCGCTATGAAGTTCC-3′	138
	(R) 5′-GTTGGGAGTGGTATCCTCTGTGA-3′	

**Table 2:** Primers of rat for real-time RT-PCR.

Gene	Sequences	Length (bp)
*β-actin*	(F) 5′-GTCAGGTCATCACTATCGGCAAT-3′	147
	(R) 5′-AGAGGTCTTTACGGATGTCAACGT-3′	
*iNOS*	(F) 5′-CACCCAGAAGAGTTACAGC-3′	186
	(R) 5′-GGAGGGAAGGGAGAATAG-3′	
*COX-2*	(F) 5′-AGAGTCAGTTAGTGGGTAGT-3′	170
	(R) 5′-CTTGTAGTAGGCTTAAACATAG-3′	
*TNF-α*	(F) 5′-CCACGCTCTTCTGTCTACTG-3′	145
	(R) 5′-GCTACGGGCTTGTCACTC-3′	
*IL-1β*	(F) 5′-TGTGATGTTCCCATTAGAC-3′	131
	(R) 5′-AATACCACTTGTTGGCTTA-3′	
*IL-6*	(F) 5′-AGCCACTGCCTTCCCTAC-3′	156
	(R) 5′-TTGCCATTGCACAACTCTT-3′	

## Abbreviations

Lico.A	Licochalcone A
PD	Parkinson’s disease
LPS	Lipopolysaccharide
SN	Substantia nigra
SNpc	Substantia nigra pars compacta
TH	Tyrosine hydroxylase
Iba-1	Ionized calcium-binding adaptor molecule-1
